# Supplementary figures and images for: Bayesian calibration, process modeling and uncertainty quantification in biotechnology
Source: PLoS Comput Biol. 2022 Mar 7;18(3):e1009223. doi: 10.1371/journal.pcbi.1009223 (PMC8939798; doi:10.1371/journal.pcbi.1009223)

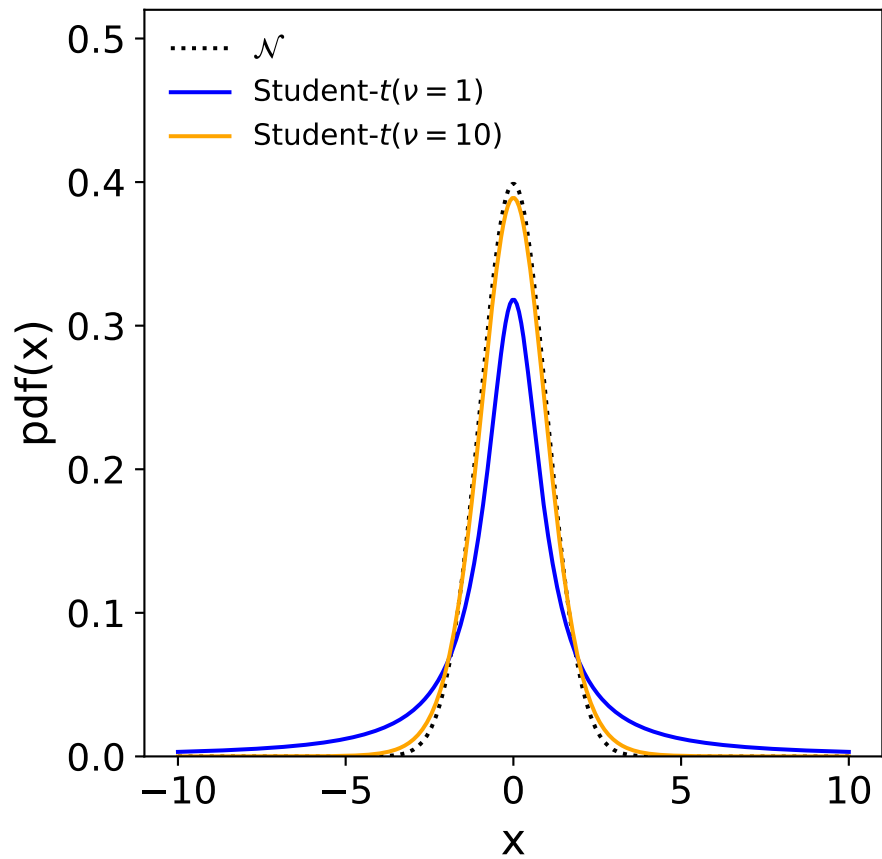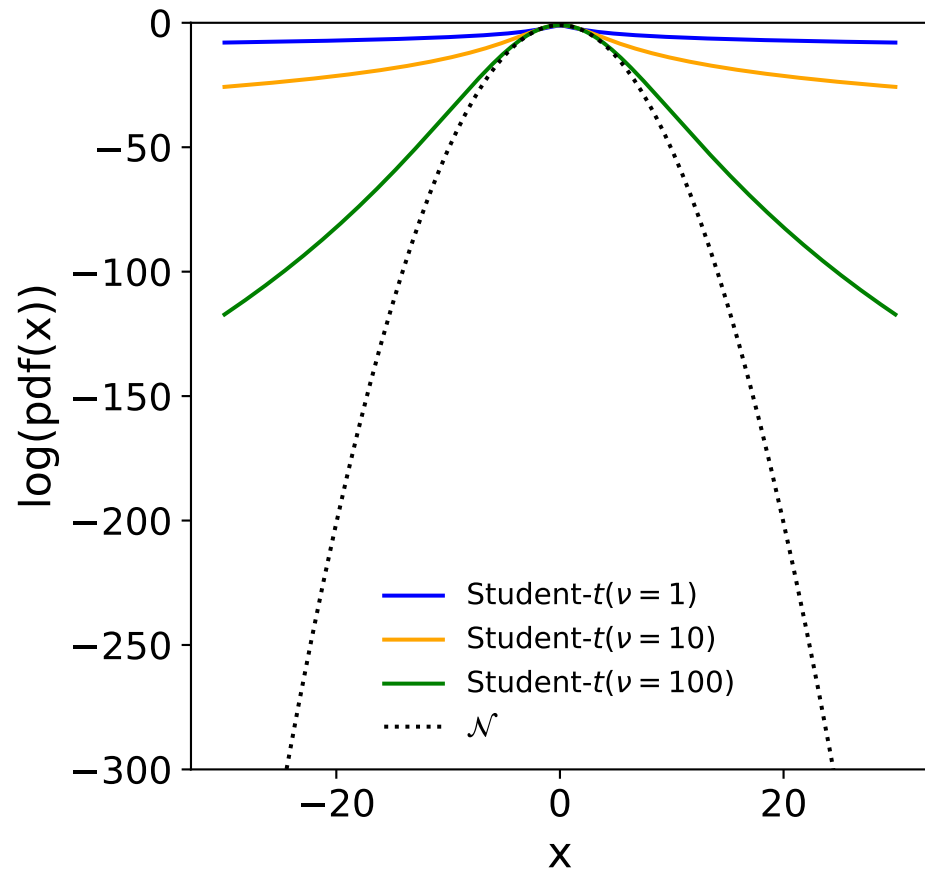

Supplement: S1 Fig — In the left chart, the probability density function (PDF) of a Normal distribution, as well as two Student-t distributions with varying degree of freedom (ν) are shown. Both distributions are parametrized by a location parameter μ that is equal to the mean and mode of these distributions. In addition to μ, the Normal is parametrized by its standard deviation parameter σ, influencing the spread of the distribution. In contrast, the Student-t distribution has two spread parameters {scale, ν} and is characterized by more probability mass in the tails of the PDF, not approaching 0 as quickly as the PDF of the Normal. With increasing ν, the Student-t distribution becomes more similar to the Normal distribution. The log probability density (right) of the Normal distribution accelerates has a quadratic dependency on the distance to the mean, whereas the log-PDF of the Student-t distribution does not go to extreme values as quickly. Because of this property, the Student-t distribution causes less numerical problems at extreme values. (PDF) [file pcbi.1009223.s001.pdf]

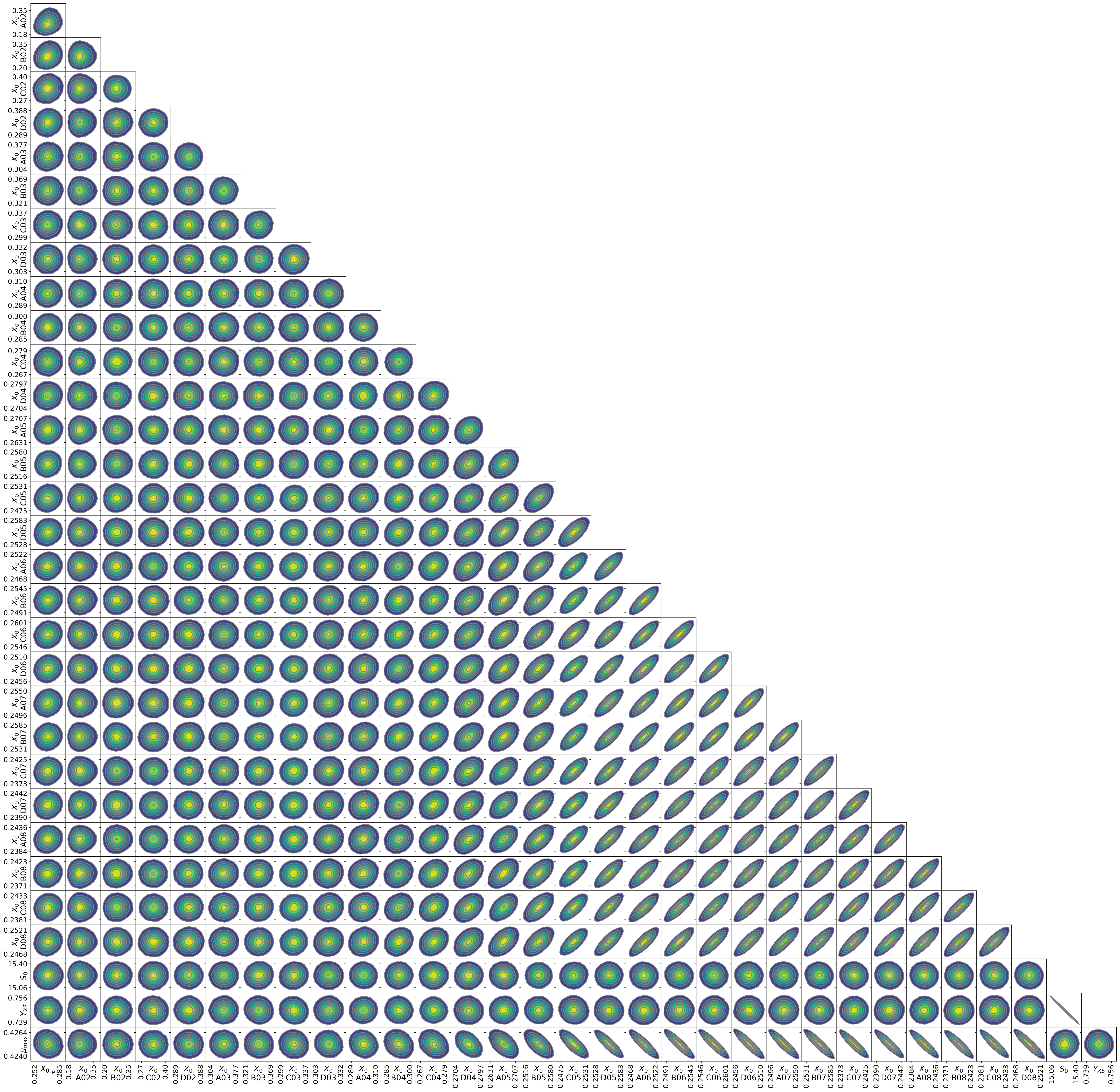

Supplement: S2 Fig — Axis labels mark the 90% HDI and subplot axis limits are set at the 98% HDI. Units are h−1 for μmax, gglucosegbiomass for YXS and gL for S0 and X0. (PDF) [file pcbi.1009223.s002.pdf]
