# Supplementary material for: Bayesian calibration, process modeling and uncertainty quantification in biotechnology
Source: PLoS Comput Biol. 2022 Mar 7;18(3):e1009223. doi: 10.1371/journal.pcbi.1009223 (PMC8939798; doi:10.1371/journal.pcbi.1009223)
Supplement: S1 File — (PDF) [file pcbi.1009223.s003.pdf]

Reparametrization of asymmetric logistic function

For simplicity of the reparameterization, the asymptote parameters L_L and L_U of the original Richard's Curve (Eq 1) can be omitted and substitutions $b = -B$ and $1/v = -e^{-c}$ were made such that all parameters may be real numbers (Eq 2).

$$\begin{aligned} f(x) &= L_L + \frac{L_U - L_L}{(1 + e^{-B(m-x)})^{1/v}} \\ L_L, L_U, B, m &\in \mathbb{R} \\ v &\in \mathbb{R}_{>0} \end{aligned} \tag{1}$$

In (3) the symmetry is already parametrized exactly as in the final result ((9)): As it increases, the inflection point I_y moves towards the upper limit. At $c = 0$ it lies centered between the limits.

$$\begin{aligned} f(x) &= \frac{1}{(1 + e^{-b(a-x)})^{e^{-c}}} \\ a, b, c &\in \mathcal{R} \end{aligned} \tag{2}$$

In the following steps a and b are reparametrized in terms of the x-coordinate of the inflection point I_x and the slope S at the inflection point respectively. I_x was obtained by solving the second derivative of the a, b, c parametrization in Eq 2 for a in Eq 3:

$$\begin{aligned} & f''(I_x) = 0 \\ \Leftrightarrow \quad & I_x = a - \frac{c}{b} \\ \Leftrightarrow \quad & a = I_x + \frac{c}{b} \end{aligned} \tag{3}$$

The slope parameter was obtained by substituting x in the first derivative of the a, b, c parametrization in Eq 2 with the analytical solution for I_x from Eq 3.

$$\begin{aligned} & S = f'(I_x) \\ \Leftrightarrow \quad & S = b(e^c + 1)^{-1-e^{-c}} \end{aligned} \tag{4}$$

Substituting a in Eq 2 with $a(I_x, b, c)$ from Eq 3 yields a parametrization in terms of I_x, b, c (Eq 5):

$$\begin{aligned} f(x) &= (e^{b(I_x - x + \frac{c}{b}) + 1})^{-e^{-c}} \\ I_x, b, c &\in \mathcal{R} \end{aligned} \tag{5}$$

For a parametrization in terms of both I_x and S, their equations from Eq 3 and Eq 4 must be solved for a and b:

$$\begin{aligned} a &= \frac{I_x e^c}{e^c + 1} + \frac{I_x}{e^c + 1} + \frac{c(e^c + 1)^{-1-e^{-c}}}{S} \\ b &= S(e^c + 1)^{(e^c + 1)e^{-c}} \end{aligned} \tag{6}$$

A parametrization in terms of I_x, S, c is then obtained by substitution of a an b in Eq 2:

$$
\begin{aligned}
f(x) &= (e^{(e^c+1)^{(e^c+1)e^{-c}} \cdot (I_x S - Sx + c(e^c+1)^{-(e^c+1)e^c})} + 1)^{-e^{-c}} \\
I_x, S, c &\in \mathcal{R}
\end{aligned} \tag{7}
$$

By common subexpression elimination Eq 7 simplifies to Eq 8.

$$
\begin{aligned}
f(x) &= (e^{x_2 \cdot (I_x S - Sx + \frac{c}{x_2})} + 1)^{x_1} \\
x_0 &= e^c + 1 \\
x_1 &= e^{-c} \\
x_2 &= x_0^{x_0 \cdot x_1} \\
I_x, S, c &\in \mathcal{R}
\end{aligned} \tag{8}
$$

The final generalized parametrization in Eq 9 in terms of L_L, L_U, I_x, S, c was obtained by scaling slope parameter and function value with $L_U - L_L$ and shifting by L_U.

$$
\begin{aligned}
f(x) &= L_L + \frac{L_U - L_L}{(e^{s_2 \cdot (s_3 \cdot (I_x - x) + \frac{c}{s_2})} + 1)^{s_1}} \\
s_0 &= e^c + 1 \\
s_1 &= e^{-c} \\
s_2 &= s_0^{(s_0 \cdot s_1)} \\
s_3 &= \frac{S}{L_U - L_L} \\
L_L, L_U, I_x, S, c &\in \mathbb{R}
\end{aligned} \tag{9}
$$

The corresponding Python implementation is shown in Code 1. For a step by step derivation of Eq 9, as well as its inverse using `sympy` we refer to the "Background Asymmetric Logsitc" Jupyter notebook in the `calibr8` repository [1].

Code 1. Implementation of reparameterized asymmetric logistic function.

```
def asymmetric_logistic(x, theta):
    """5-parameter asymmetric logistic model.

    Parameters
    ----------
    x : array-like
        independent variable
    theta : array-like
        parameters of the logistic model
            L_L: lower asymptote
            L_U: upper asymptote
            I_x: x-value at inflection point
            S: slope at the inflection point
            c: symmetry parameter (0 is symmetric)

    Returns
    -------
    y : array-like
        dependent variable
    """
    L_L, L_U, I_x, S, c = theta[:5]
    # common subexpressions
    s0 = numpy.exp(c) + 1
    s1 = numpy.exp(-c)
    s2 = s0 ** (s0 * s1)
    # re-scale the inflection point slope with the interval
    s3 = S / (L_U - L_L)

    x = numpy.array(x)
    y = (numpy.exp(s2 * (s3 * (I_x - x) + c / s2)) + 1) ** -s1
    return L_L + (L_U-L_L) * y
```

References

1. Osthege M, Helleckes L. JuBiotech/calibr8: v6.2.0; 2021. Available from: https://doi.org/10.5281/zenodo.5721015.
