# Supplementary material for: Bayesian calibration, process modeling and uncertainty quantification in biotechnology
Source: PLoS Comput Biol. 2022 Mar 7;18(3):e1009223. doi: 10.1371/journal.pcbi.1009223 (PMC8939798; doi:10.1371/journal.pcbi.1009223)
Supplement: S2 File — (PDF) [file pcbi.1009223.s004.pdf]

Mathematical notation of calibration models

The linear glucose calibration model uses a 1st order polynomial of the glucose concentration x to describe the location parameter of a Student-t distribution. The scale parameter was modeled as a 1st order polynomial function of the location and the distributions degree of freedom ν was estimated as a constant.

$$\begin{aligned}
\mathrm{A}_{360\ \mathrm{nm}} &\sim \text{Student-}t(\mathrm{loc}, \mathrm{scale}, \nu) \\
&\text{with} \\
\mathrm{loc} &= \mu_0 + \mu_1 x \\
\mathrm{scale} &= \mathrm{loc} \cdot \mathrm{scale}_1 + \mathrm{scale}_0 \\
\mu_0, \mu_1, \mathrm{scale}_0, \mathrm{scale}_1 &\in \mathbb{R} \\
\nu &\in \mathbb{R} > 0
\end{aligned} \tag{1}$$

In comparison to the linear version in Eq 1, the logistic glucose calibration model in Eq 2 uses the asymmetric logistic function (S1 File) to describe the location parameter.

$$\begin{aligned}
\mathrm{A}_{360\ \mathrm{nm}} &\sim \text{Student-}t(\mathrm{loc}, \mathrm{scale}, \nu) \\
&\text{with} \\
s_0 &= -L_L + L_U \\
s_1 &= e^c + 1 \\
s_2 &= e^{-c} \\
s_3 &= s_1^{s_1 s_2} \\
\mathrm{loc} &= L_L + s_0 \left(e^{s_3 \left(\frac{S(I_x - x)}{s_0} + \frac{c}{s_3} \right)} + 1 \right)^{-s_2} \\
\mathrm{scale} &= \mathrm{loc} \cdot \mathrm{scale}_1 + \mathrm{scale}_0 \\
L_L, L_U, I_x, S, c, \mathrm{scale}_0, \mathrm{scale}_1 &\in \mathbb{R} \\
\nu &\in \mathbb{R} > 0
\end{aligned} \tag{2}$$

Similar to the logistic glucose calibration model (Eq 2), the biomass calibration model in Eq 3 uses an asymmetric logistic function, but first applies a base-10 logarithm to the biomass concentration x. Due to the base-10 logarithm, the intercept and slope parameters ($I_{\log_{10}(x)}, S_{\log_{10}}$) of the biomass calibration model refer to the log scale.

$$
\begin{aligned}
\text{backscatter} &\sim \text{Student-}t(\text{loc}, \text{scale}, \nu) \\
\text{with} & \\
s_0 &= -L_L + L_U \\
s_1 &= e^{c} + 1 \\
s_2 &= e^{-c} \\
s_3 &= s_1^{s_1 s_2} \\
\text{loc} &= L_L + s_0 \left(e^{s_3 \left(\frac{S_{\log_{10}} \left(I_{\log_{10}(x)} - \log_{10}(x) \right)}{s_0} + \frac{c}{s_3} \right)} + 1 \right)^{-s_2} \\
\text{scale} &= \text{loc} \cdot \text{scale}_1 + \text{scale}_0 \\
L_L, L_U, I_{\log_{10}(x)}, S_{\log_{10}}, c, \text{scale}_0, \text{scale}_1 &\in \mathbb{R} \\
\nu &\in \mathbb{R} > 0
\end{aligned}
\tag{3}
$$
