# Supplementary material for: Bayesian calibration, process modeling and uncertainty quantification in biotechnology
Source: PLoS Comput Biol. 2022 Mar 7;18(3):e1009223. doi: 10.1371/journal.pcbi.1009223 (PMC8939798; doi:10.1371/journal.pcbi.1009223)
Supplement: S3 File — (PDF) [file pcbi.1009223.s005.pdf]

Implementation, planning and saving of calibrations

Code 1. Convenience class `BaseAsymmetricLogisticT`.

```
class BaseAsymmetricLogisticT(BaseModelT):
    def __init__(
        self, *,
        independent_key:str, dependent_key:str,
        scale_degree:int=0,
        theta_names: Optional[Tuple[str]]=None,
    ):
        """ Template for a model with asymmetric logistic trend (mu)
        and polynomial scale (as a function of mu).

        Parameters
        ----------
        independent_key : str
            name of the independent variable
        dependent_key : str
            name of the dependent variable
        scale_degree : optional, int
            degree of the polynomial model
            describing the scale as a function of mu
        theta_names : optional, tuple of str
            may be used to set the names of the model parameters
        """
        self.scale_degree = scale_degree
        if theta_names is None:
            theta_names = tuple("L_L,L_U,I_x,S,c".split(",")) + tuple(
                f"scale_{d}"
                for d in range(scale_degree + 1)
            ) + ("df",)
        super().__init__(independent_key, dependent_key, theta_names=theta_names)
```

Code 2. Human-readable pipetting instructions for the serial dilution of biomass for the calibration experiment.

```
Serial dilution plan (0.00102 to 1.00) from at least 12232.0 L stock and 54368.0 μL diluent:
  Prepare column 1 with [2000. 1727. 1491. 1287. 1111.  959.] μL from stock and fill up to 2000 μL
  Prepare column 2 with [538. 465. 401. 346. 299. 258.] μL from stock and fill up to 1300 μL
  Prepare column 3 with [223. 192. 166. 143. 124. 107.] μL from stock and fill up to 1300 μL
  Prepare column 4 with [92. 80. 69. 59. 51. 44.] μL from stock and fill up to 1300 μL
  Prepare column 5 with [39. 39. 39. 39. 39. 39.] μL from column 1 and fill up to 1300 μL (1 serial dilutions)
  Prepare column 6 with [39. 39. 39. 39. 39. 39.] μL from column 2 and fill up to 1300 μL (1 serial dilutions)
  Prepare column 7 with [39. 39. 39. 39. 39. 39.] μL from column 3 and fill up to 1300 μL (1 serial dilutions)
  Prepare column 8 with [39. 39. 39. 39. 39. 39.] μL from column 4 and fill up to 1300 μL (1 serial dilutions)
```

Code 3. JSON file containing stored model properties.

```
{
    "calibr8_version": "6.0.0",
    "model_type": "models.LogisticGlucoseCalibrationModelV1",
    "theta_names": [
        "L_L", "L_U", "I_x", "S", "c", "scale_0", "scale_1", "df"
    ],
    "theta_bounds": [
        [-Infinity, 0.3],
        [2, 5],
        [-50, 50],
        [0, 20],
        [-3, 3],
        [0, 0.1],
        [0, 0.06],
        [1, 20]
    ],
    "theta_guess": [0.1, 2.8, 1.2, 10, 1, 0.08, 0.01, 3],
    "theta_fitted": [
        -8.812, 2.765, 8.246, 0.0839,
        2.69, 0.000374, 0.0154, 3.007
    ],
    "theta_timestamp": "2021-02-15T14:27:11Z",
    "independent_key": "glc",
    "dependent_key": "A365",
    "cal_independent": [
        50.0,
        27.94736842105263,
        ...
        0.05030330952033825
    ],
    "cal_dependent": [
        2.6449,
        2.2389,
        ...
        0.1166
    ]
}
```
