# Supplementary material for: Bayesian calibration, process modeling and uncertainty quantification in biotechnology
Source: PLoS Comput Biol. 2022 Mar 7;18(3):e1009223. doi: 10.1371/journal.pcbi.1009223 (PMC8939798; doi:10.1371/journal.pcbi.1009223)
Supplement: S4 File — (PDF) [file pcbi.1009223.s006.pdf]

Process model parametrization

The parametrization of the batch cultivation process model was given by the tabular notation of a `murefi.ParameterMapping` in Table 1.

Table 1. Parameter mapping for fitting of Monod kinetics.
Repetitive rows were left out for clarity. The full length table has 28 rows.

replicate	S_0	X_0	μ_{max}	K_S	Y_{XS}
A02	`S0`	`X0_A02`	`mu_max`	0.02	`Y_XS`
...	`S0`	`X0_...`	`mu_max`	0.02	`Y_XS`
A08	`S0`	`X0_A04`	`mu_max`	0.02	`Y_XS`
...	`S0`	`X0_...`	`mu_max`	0.02	`Y_XS`
D08	`S0`	`X0_D08`	`mu_max`	0.02	`Y_XS`
