# Supplementary material for: Bayesian calibration, process modeling and uncertainty quantification in biotechnology
Source: PLoS Comput Biol. 2022 Mar 7;18(3):e1009223. doi: 10.1371/journal.pcbi.1009223 (PMC8939798; doi:10.1371/journal.pcbi.1009223)
Supplement: Attachment — Submitted filename: response.pdf [file pcbi.1009223.s008.pdf]

calibr8 Review Response

Comment to the Editor and Reviewers

Thank you very much for sending the reviewers´ comments. We would like to thank the reviewers for their positive feedback and constructive suggestions, which led to changes in the manuscript. The changed sections in the revised manuscript are highlighted in green color. We hope that we could increase the quality of the manuscript and that it is now acceptable for publication.

Reviewer 1

> Helleckes et al describe a computational framework for the Bayesian analysis of calibration and process models. The framework is implemented in python, open-source, extensively documented implements code in a object-oriented manner. The software is tested using continues integration and coverage analysis. Overall I want to commend the authors for the quality of the presented software. The manuscript is well written and easy to understand, but may benefit from some restructuring that I will describe below. The authors evaluate the framework on multiple examples of biotechnological processes, including experimental data, where they analysis calibration models individually, as well as combinations of calibration and process models.

Major Comments

> 1. While most of the text is well written and easy to follow, there are some instances where it is difficult or impossible to understand the text without looking at the code or figures. Examples:

> (i) Figure 3: Description of what was done is only available in figure legends, this also needs to be described in the text.

Response: We added more descriptions of what is shown in the figure to the text.

> (ii) 4.1.1: It would helpful to have a mathematical description of the calibration model (provide formulas)

Response: We have added mathematical notations in the appendix.

> (iii) Figure 10: It is hard to understand what was actually done and the reader has to guess based on the figure. Describe what was done first, then the results.

Response: We added further explanations in the text and added a reference to the respective methods chapter, in which a detailed explanation of the `infer_independent` method can be found.

> 2. The (unpublished) python implementation of PESTO, pyPESTO https://github.com/ICB-DCM/pyPESTO), implements a similar feature set as the murefi/calibr8 including providing an interface to pyMC3 for Bayesian analysis. Still pyPESTO implements less modularity between process model and calibration model to enable use of adjoint sensitivity analysis.

Response:

- pyPesto is working with AMICI models (interface for two different SUNDIALS solvers) and recently started featuring Aesara objectives
- Direct import of ODEs is not supported in AMICI, so users need to go the route via SBML or PETab and implement the Hessian and derivative
- If users install SUNDIALS, they get the gradients in calibr8 / murefi as well
- Greater modularity is achieved in calibr8 and murefi since new ODEs can be easily implemented in murefi without the need to implement further functions and independently of other frameworks
- calibr8 allows for more customization in the noise models and to independently look at the calibration process rather than binding it to the overall process model
- For compatibility to pyPESTO, a murefi objective could be inserted as an Aesara objective if desired.
- pyPESTO was entered in the comparative table and included in the discussion

> Similarly, the recently proposed PEtab format (https://journals.plos.org/ploscompbiol/article?id=10.1371/journal.pcbi.1008646) permits a similarly flexible specification of calibration models and multi-experiment process models (templating) in COPASI/data2dynamics/pyPESTO. Accordingly, I think it would be important to
> (i) explicitely describe differences to what is possible with PEtab
> (ii) demonstrate the greater flexibility by the modularity between calibr8 and murefi.

Response: Regarding PETab in general:

- Development of murefi and calibr8 was mainly done when PETab did not exist. However, we recognize the potential of the new data format.
- Currently, the biggest limitations of PETab in comparison to our framework are the restriction to Laplace or Normal distribution as noise models ([PETab Docs (https://petab.readthedocs.io/en/latest/documentation_data_format.html#overview)](https://petab.readthedocs.io/en/latest/documentation_data_format.html#overview)). As shown by the examples in our documentation, `calibr8` is not limited to any particular univariate or multivariate noise distribution.
- Moreover, PETab so far does not support hierarchical calibration models using hyperpriors shared between different calibration model instances. With `calibr8` or `murefi` this can be easily achieved because they are compatible with the Theano/Aesara/PyMC modeling framework.
- We thus think that `calibr8` allows greater modularity and a broader application

spectrum, also by providing functions like saving, loading and combining various calibration models for analysis.

- Exporting `calibr8` as PETab noise models could be useful if further modeling efforts are constrained to take PETab inputs. However, `calibr8` models may contain rather involved "noise formulas", take multivariate inputs, or use noise distributions not implemented by PETab. We think this is a general limitation of text-based model specification formats including PETab and SBML, which is one reason why model implementations in `calibr8` and `murefi` are kept as source code.
- These points are now also adressed in section 4.3 (Comparison to existing software) of the article.

> Regarding (i), I would also encourage the authors to implement support for the PEtab format and potentially propose an extension of the format based on the identified differences (I don't think these points are necessary for the scope of this review though)

Response: See previous response and text modifications. As suggested by the reviewer, implementation of PETab support is out-of-scope for this article though.

> Regarding (ii) I think the use of a hyperprior in Section 4.2.3 is an interesting example, but I think it would be more convincing if the nesting would be in the calibration model component and if the authors could demonstrate that joint analysis of process+calibration model is important to obtain accurate credibility intervals for parameters (by comparing stepwise sequential analysis, using synthetic data if necessary)
> Am I correct in my understanding that the toolbox cannot be applied to mixed effect modeling? If I am mistaken, this would be quite convincing and a demonstration could replace point (ii).

Response: Thank you for these comments. The classification "mixed effect model" is rather diffuse, but to our understanding, both the ODE model in the manuscript (X_0 hyperprior) and the [example we added to the `calibr8` documentation](https://calibr8.readthedocs.io/en/latest/Advanced_Hierarchical_Calibration.html) (https://calibr8.readthedocs.io/en/latest/Advanced_Hierarchical_Calibration.html) (nested intercept, σ parameters) fall into the category of nonlinear mixed effect models.
The example in the documentation also jointly estimates calibration and process model parameters, which we did not do in the analysis for this manuscript since the biomass calibration model was well informed by the data.
While the example was written with a `NormalNoise` model to make it more accessible, more complex noise models with LogNormal, Laplace, or even discrete distributions such as Poisson are perfectly compatible.

> A third way of addressing (ii) would be to demonstrate that the combination of calibr8/murefi/sunode enables the use of adjoint sensitivities (see https://journals.plos.org/ploscompbiol/article?id=10.1371/journal.pcbi.1005331) with complex noise models, but I expect that this would be a lot of work
>
> 3. The description in 3.2.6 seems to imply that murefi supports sensitivity analysis which would be necessary for the computation of the posterior gradient. Looking at the code, this does not seem to be supported though, but theoretically possible with sunode. It would be helpful if the authors could more extensively describe the support of gradient computation in calibr8/murefi.

Response: Yes, in general gradients can be obtained via the Theano/Aesara backends. The `IntegrationOp` in murefi does not implement a `.grad()` itself, since `sunode` already provides an excellent implementation of Theano/Aesara compatible ODE differentiation methods. Under https://murefi.readthedocs.io/en/latest/Example_04_MichaelisMenten_sunode.html the `murefi` documentation includes an example of gradients with `sunode`. Gradients of simpler non-ODE models can be obtained the same way without `sunode`.
Our call to `sunode's solve_ivp` (https://github.com/JuBiotech/murefi/blob/v5.0.1/murefi/symbolic.py#L160) specifies the use of forward sensitivities, because it's more efficient for small systems, but since `sunode` also provides adjoint sensitivities, we will consider to forward user-specified kwargs to make the `derivatives` strategy user-configurable.

Minor Comments

> a) I believe that the abstract should mention that the authors apply their methods to ODE models.

Response: Adressed in lines 21-25.

> b) l244, the text just mentions calibr8, which is only introduced later in the text

Response: calibr8 and murefi are now both mentioned in aim of the study

> c) I have a hard time understanding the issue that is supposed to be illustrated by figure 3C, could the authors describe the issue in more detail, is there some statistical test to show the issue (and is such an analysis implemented in calibr8)?

Response: The lack-of-fit of the noise model is indeed hard to spot. We have added additional explanations in the text, alongside a reference to Kolmogorov-Smirnov tests, and recent work on obtaining ECDF confidence intervals specifically for such diagnostic visualizations.

> d) I find it hard to believe that gradient based optimizers did not perform well for section 3.2.5. Did the authors try using the least squares algorithm and used a logarithmic transformation of process parameters?

Response: We revised the paragraph since gradient-based optimizers are possible in general. We currently have an open issue (https://github.com/JuBiotech/calibr8/issues/13) regarding robustness of the implementation and will see how we can further improve it.

> e) the authors may want to cite https://doi.org/10.1093/bioinformatics/btw703 in section 2.4

Response: Thank you for pointing out this source. We included it in the respective section. Laplace distributions are also available now in calibr8 as one default option.

> f) I think "Frequentist" is more commonly used than "likelihoodist" (assuming that's what the authors wanted to say).

Response: We agree that frequentist is the more commonly used term. However, we explicitly wanted to make the differentiation between likelihoodist and frequentist.

Reviewer 2

> This proposal is focused on the description of a Python package for the use of Bayesian theory for the quantification of uncertainty from high-throughput cultivation experiments. The paper is technically sound, well written and all the codes are available on a Git server. Instructions about the installation on a Python idle are clearly given.This proposal has been submitted as a "software" article. As such, the software must be either widely used within the scientific community or have the promise of wide adoption by a broad community of users. However, as it is actually written, the target audience is quite limited. Specific comments are appended below.

Major

Response: We appreciate the reviewer´s advise, since we aim to address a broad community. The presented software is not limited to microbioreactor data or any specific measurement technique. However, our examples come from the microbial bioprocess area, since it is a main interest for our research group. To open the text to a wider community, we revised the manuscript and tried to avoid specific technical terms that might limit the accessibility. Although applications even beyond biotechnology are possible, we addressed this area in the title to set a clear scope for this article.

- The background needs to be more precisely defined. The field of expertise of the authors are “bioprocess engineering”. However, as stated in the introduction, the software could be used by any researchers handling large set of microbial (or even cell culture) kinetics data, extending the target audience to the broad community if researchers involved in fields like systems and synthetic biology. Some part of the text should then be reformulated according to this comment.

Response: We revised the text to simplify the terminology where possible to target a broad community. Together with the new examples added to the manuscript and the documentation, we aimed at visibly broadening the target audience.

- The term “microbioreactor” is used at several stages of the manuscript. This term is actually misleading, because the authors are used a commercial minibioreactor platform based on the use of deepwell. For the readers that are not familiar with the technology, the use of the term “micro” could give the feeling that the authors are using microfluidic cultivation device. This comment is also related to the above-mentioned in the sense that the technical terms in the text should be reformulated to target a broader audience of potential users (other example: I can imagine that only a limited number of specialists know what is a flowerplate).

Response: The term microbioreactor (MBR) is commonly used for cultivation devices, such as the BioLector. Moreover, a review on MBR systems by Hemmerich *et. al* 2018 (https://doi.org/10.1002/biot.201700141) compares different systems and lists the mentioned device as a typical microbioreactor.
To the best of our knowledge, the term mini bioreactor usually refers to small stirred reaction systems with volumes in the range of several milliliter (e.g. the 2mag, ambr or DASbox systems). However, all publications we found using the BioLector use the term microbioreactor. To keep to the commonly used nomenclature, we kept the term microbioreactor.
However, we tried to revise the text concerning bioprocess-engineering-specific jargon to keep the accessibility for users who are not familiar with the technology.

- At this stage, the applicability of the software is limited to a cultivation device commercialized under the name of “Biolector” and involving the use of 48-well deepwell plates. Additionally, the Biolector can be coupled to a robotic liquid handling platform for off-line sampling. The authors have to discuss the potential extension of their software to other microplate-based devices.

Response: While our application example in this study used a microcultivation device, our software is completely agnostic to the field of application. Especially the `calibr8` package is purely concerned with the statistical properties of calibration models.
Our `murefi` package is specifically designed around multi-replicate (multi-level) experiments that are to be modeled with ordinary differential equations, but again is agnostic to the field of application.

Both packages are designed to take standard data structures from the scientific Python ecosystem (NumPy arrays, pandas DataFrames) as inputs, thereby enabling users to apply them to any kind of experimental dataset.
We also pointed this out in the manuscript after the implementation of the first two calibration models (end of section 4.1.2).
Further application examples such as ODE modeling of catalytic reactions can be found in the documentation.

> - Bayes theory is typically easy to explain, but it is not the case of this paper. Section 2.1 should be expanded by considering an example.

Response: After moving the Normal/Student-*t* comparison to the appendix, Section 2.1 now includes a visual explanation of Bayes' rule for continuous variables. Furthermore, we refined the equations with subscripts and colors to help beginners to connect the mathematical notation with the visual representation. We also changed the order of equations to start with Bayes' theorem, which might be more familiar to the reader.

> - About the applications, only two are given in the manuscript. Another, very important, application is the measurement of the activity of gene circuits based on fluorescent reporters. For this application, it is also important to relate to biomass in order to obtain specific values. How could this application integrated in the package ?

Response: Assuming a photometric measurement system: First we recommend to scan absorbance spectra of individual sample components to determine a wavelength at which biomass may be observed with the least interference. Second, a dilution series of biomass should be measured at the selected wavelength to construct a biomass/absorbance calibration model. After measuring these wavelengths together with the fluorescences, one may apply the calibration model to infer sample-wise biomass concentrations. If you also have a process model to describe progression of fluorescences, the calibration model may be included as the "observation function" for the corresponding biomass concentration.
While we don't want to cover this specific application in the manuscript, we'd be happy to discuss this application in a GitHub issue.

> - ODE models can be integrated in the calibration procedure. Is there any limit about the number of equations/parameters that can be handled ?

Response: We are currently not aware of technical limitations from `murefi` or `sunode`. In practice a limitation may depend on the number of equations & parameters in the model, as well as the CPU & memory resources available. Tens of equations or replicates are absolutely fine.
While we think that benchmarking and improvement of the computational performance would be a valuable contribution to the `sunode` and `murefi` projects, we believe that it is out-of-scope for this article.

Reviewer 3

In the manuscript "Bayesian calibration, process modeling and uncertainty quantification in biotechnology", Laura Marie Helleckes, Michael Osthege, and coworkers present two Python software packages, calibr8 and murefi, for enabling more reproducible and automated calibration of mathematical models in biotechnology. The authors show the capabilities of these packages on several examples with real datasets collected by themselves.

The topic of parameter inference for mathematical models of biological systems have become a very relevant topic in the recent years. This can be achieved by a frequentist perspective, finding the maximum likelihood estimate using various optimization techniques, or by a Bayesian approach, which relies on the Bayes theorem and often carried through Markov chain Monte Carlo sampling.

One of the key problems is that frequently “handmade” computational pipelines are built for each specific problem which compromises their reproducibility and reusability. Efforts in the community to build automated pipelines exist, however these can be complex and require high expertise. In this manuscript, the authors tackle this problem by introducing two new software tools for model construction and calibration, which substantially ease the process and facilitate the usage to non-experts, in particular, focusing - but not limited to - biotechnology applications. Likewise, the key contributions is the development of two reusable open-source toolboxes/libraries. Overall, the manuscript is well written. I appreciate that the authors provide exemplary notebooks/code in the respective toolboxes, but I have to admit that I did not have the time to test them.

Major

As far as I can see, the only available loglikelihood implemented in calibr8 assumes a Student’s t distribution. This is a bit contradictory with the statement the authors make in L.67-69 regarding the need of having more flexible frameworks. Maybe the authors could include in their toolbox additional distribution assumptions such as Laplace, Gaussian and/or log-normal, to facilitate the users this flexibility.
Moreover, I do not see how the user could easily implement a different custom noise model, in case this is possible it would be great to add a tutorial.

Response: Inspired by your concern, we have refactored the class inheritance in `calibr8` `v6.2.0` such that now the `loglikelihood` implementation is completely agnostic to the noise model.
An example for how to implement custom noise models [was added to the documentation (https://calibr8.readthedocs.io/en/latest/Advanced_Custom_Noise_Models.html)](https://calibr8.readthedocs.io/en/latest/Advanced_Custom_Noise_Models.html).

> A very well-known standard format to encode ODE models is the SBML format (https://doi.org/10.1093/bioinformatics/btg015) which is supported by many existing toolboxes for modeling and parameter estimation. Including support to this in the toolboxes here presented would substantially increase the public and potential new users to the tool. I deeply encourage the authors to add support to SBML models although not only ODE models are used within these toolboxes.

Response: The `murefi.BaseODEModel` requires primarily the vector of independent variable names, and a `dydt(y, t, ode_parameters)` function implementing the differential equations. This information could originate from a variety of sources, including SBML, PETab or similar formats. We considered the option to implement a `murefi.contrib.sbml_import` module, but given the apparent complexity (the AMICI `sbml_import.py` module, for example, has 2x more lines than all of `murefi`) we decided against maintaining a SBML import module. If the reviewer can point us to a concise code example on how species names and a `dydt()` function can be imported, optionally using a lightweight 3rd party dependency, we are happy to add an example to our documentation.

> Following on the line of my previous comment: Is there a model validator? What I mean is whether is there some sort of sanity checks for user defined models in calibr8 and murefi., e.g. positivity of the modeled species. This would make even better the user experience, since then, even the level of expertise required could be lowered. I am wondering if the authors thought of this option, and whether it would be possible to include it (or at least comment on this).

Response: This kind of model validation would also require the user to input such constraints, rendering the API much more verbose. Instead we recommend to use the `predict_replicate` method during model development and visualize model predictions from realistic parameter vectors such as the initial guess of the parameter estimation, or to draw prior predictive samples when working inside a PyMC model. For `calibr8`, diagnostics are also detailed in Section 2.5.

> L.88: “examples are Data2Dynamics [14] or PESTO [15], ... However, both tools are implemented in MATLAB and are thus incompatible with data analysis workflows that leverage the rich ecosystem of scientific Python libraries.” I would like to make the authors aware that I could find that the toolbox PESTO has been translated into Python, going under the name of pyPESTO (https://github.com/ICB-DCM/pyPESTO). I would encourage the authors to include it into their manuscript since it has been released since January 2019 and, therefore, adapt the comparison within toolboxes.

Response:
Has been addressed in Table 1 as well as the respective text (also see comments for reviewer 1 for detailed changes).

> L.177: The statement is correct. However, parameter uncertainties can also be quantified from a frequentist perspective using optimization by the so-called method profile likelihoods (see https://doi.org/10.1093/bioinformatics/btp358). I am not aware whether this is known in the field of biotechnology, but definitely something to mention in a manuscript regarding parameter estimation and uncertainty quantification. In case this is not frequently used in this field, it could be also a novelty to add in the study (although not necessary).

Response: We are aware of profile likelihoods, but for the kinds of models we are working with, MCMC sampling the joint posterior works just fine. Furthermore we are generally hesitant about the added implementation complexity of iterating over the free parameters individually and running that many optimizations. In contrast, the application of MCMC works off the shelf and a wide variety of diagnostics & visualizations are available through community packages such as ArviZ.

> L.205: Could some citation be added to this statement?

Response: Citation was added.

> L.226-227: Could some citation be added to this statement?

Response: Thank you for bringing this statement to our attention. We clarified that it is a recommendation based on this work and gave two reasons for it. Since one sentence cannot adequately summarise the current research on model selection for biotechnology and since it is not the focus of the presented software, we decided to leave out a respective statement.

> L.244: Please reformulate the sentence, I could not understand which restriction is meant here.

Response: We reformulated the sentence, pointing towards the `calibr8` documentation example where calibration model parameters are not fixed, but estimated jointly with a process model.

Minor

> L.112: Without losing generality, the authors could list some specific examples of other research fields.

Response: We have added a sentence mentioning examples from our documentation and how they can be transferred to other domains.

> Figure 1: I suggest to use the same label and line style in the two subplots for the Normal case.

Response: The figure was adapted, but moved to the supporting information due to other review comments on the chapter.

> L.142: “From a known list of parameters …” The word “known” here could lead to misunderstanding since the parameters may be actually unknown and need to be estimated. But I understand that when simulating they are actually “known”. Maybe this sentence could be rephrased (in case of finding a better formulation).

Response: Reformulated to *given list of parameters.*

> L.158: Please clarify that each individual pair y_{obs} and y_{pred} are the same length.

Response: Paragraph was changed accordingly.

> Figure 3: Please indicate in the figure as legend what the blue dots and green lines are. Having this on top of the caption description will facilitate the understanding of what is depicted. Same for the black dashed line.

Response: Figure was revised accordingly.

> Figure 7: Change the color scheme → colorblind proof

Response: One of the authors has a color vision deficiency but no problems with this figure. We changed red→blue anyway.

> L.540: To the list of known samplers, please add “emcee” which is also a very popular python sampler (https://doi.org/10.1086/670067).

Response: Added as requested.

> Figure 12: Please increase the separation between A and B. This helps to identify the right Y axis in A.

Response: Modified as suggested.

> General: Revise the text for typos.

Response: We thoroughly read through the text again and corrected typos.

> General: Please use consistent font sizes in the figures, for some, they are really small.

Response: We have increased font sizes where possible. We anticipate that further fine tuning will be done by the journal based on the vector graphic files we provide.
